# Regulatory T Cell-Targeted Immunomodulatory Therapy for Long-Term Clinical Improvement of Atopic Dermatitis: Hypotheses and Perspectives

**DOI:** 10.3390/life13081674

**Published:** 2023-08-01

**Authors:** Dong-Ho Nahm

**Affiliations:** Department of Allergy and Clinical Immunology, Ajou University School of Medicine, Suwon 16499, Republic of Korea; dhnahm@gmail.com; Tel.: +82-31-219-5152

**Keywords:** hypersensitivity, immunomodulation, allergy and immunology, immunotherapy, immune tolerance, atopic dermatitis, atopic eczema, regulatory T cell

## Abstract

Atopic dermatitis (AD) is a chronically relapsing inflammatory skin disorder characterized by itching and eczematous lesions. It is often associated with a personal or familial history of allergic diseases. Allergic inflammation induced by immunoglobulin E and T-helper type 2 (Th2) cell responses to common environmental agents has been suggested to play an essential role in AD pathogenesis. The standard therapies for AD, including topical or systemic agents, focus on controlling skin inflammation. Recently developed monoclonal antibody to interleukin-4 receptor alpha or Janus kinase inhibitors can provide significant clinical improvements in patients with AD by inhibiting Th2 cell-mediated skin inflammation. However, the clinical efficacy of the Th2 cell-targeted therapy is transient and incomplete in patients with AD. Patients with AD are seeking a permanent cure. Therefore, the development of novel immunomodulatory strategies that can improve a long-term clinical outcome and provide a long-term treatment-free clinical remission of AD (disease-modifying therapy) is needed. Regulatory T (Treg) cells play a critical role in the maintenance of immune tolerance and suppress the development of autoimmune and allergic diseases. This review provides three working hypotheses and perspectives for the treatment of AD by Treg cell activation. (1) A decreased number or function of Treg cells is a critical event that causes the activation of Th2 cells, leading to the development and maintenance of AD. (2) Activation of Treg cells is an effective therapeutic approach for AD. (3) Many different immunomodulatory strategies activating Treg cells can provide a long-term clinical improvement of AD by induction of immune tolerance. The Treg cell-targeted immunomodulatory therapies for AD include allergen immunotherapy, microbiota, vitamin D, polyvalent human immunoglobulin G, monoclonal antibodies to the surface antigens of T cell or antigen-presenting cell, and adoptive transfer of autologous Treg cells or genetically engineered Treg cells expanded in vitro.

## 1. Introduction

Atopic dermatitis (AD) is a chronically relapsing inflammatory skin disorder characterized by itching and eczematous lesions. It is often associated with a personal or familial history of allergic diseases [1,2,3]. Hypersensitivity reaction (allergic reaction) to environmental agents has been suggested as a pathogenetic mechanism responsible for the development and maintenance of chronic skin inflammation in patients with AD [3]. However, the precise pathogenetic mechanism underlying AD remains unclear. The standard therapies for AD, including topical corticosteroids or calcineurin inhibitors, focus on controlling skin inflammation [1,2,3]. A significant number of patients with AD can be further improved by systemic immunomodulatory agents including corticosteroids, cyclosporine, or methotrexate. However, there are toxicity risks associated with prolonged use of these compounds [3]. Monoclonal antibody to interleukin (IL)-4 receptor alpha and Janus kinase inhibitors suppressing T-helper type 2 (Th2) cell-mediated inflammation can provide significant clinical improvement in patients with moderate-to-severe AD [4,5,6,7]. These findings demonstrate that hypersensitivity reactions mediated by Th2 cells play a key role in the pathogenesis of AD [4,5,6,7]. These results also demonstrate that AD is a systemic immune disease, and that systemic immunomodulation is an efficient strategy for the treatment of AD. However, the Th2-targeted therapy could not modulate upstream immune dysfunction causing Th2 cell activation and could not provide a long-term clinical improvement (LTCI) of AD. Thus, further development of novel therapeutic modalities for AD that can improve a long-term clinical outcome and provide a long-term treatment-free clinical remission of AD (disease-modifying therapy) is required. Studies in patients with AD and AD mouse models have shown that immune dysfunction caused by a decreased number and/or function of regulatory T (Treg) cells is critical for Th2 cell activation and immunoglobulin E (IgE)-mediated inflammation [8,9]. Therefore, an immunomodulatory strategy that activates Treg cells is an ideal therapeutic approach to induce immune tolerance and achieve an LTCI of AD. This review provides hypotheses and perspectives on immunomodulatory strategies that activate Treg cells to achieve an LTCI of AD by induction of immune tolerance through antigen-dependent and/or antigen-independent stimulations.

## 2. Unmet Needs of Patients with AD

Patients with AD are seeking a permanent cure. This fact is supported by the persistence of various complementary and alternative therapies for AD [10], as well as multiple internet and social media contents created by patients with AD who want to share their personal experiences on successful AD self-management. However, physicians are explaining to patients suffering from AD that there is still no cure for AD and that it should be managed by continuous medical treatments [3]. This significant mismatch between patient’s needs and current medical therapies for AD necessitates further development of novel immunomodulatory strategies to achieve an LTCI of AD.

## 3. Hypothesis on the Pathogenesis of AD

**Figure 1:**
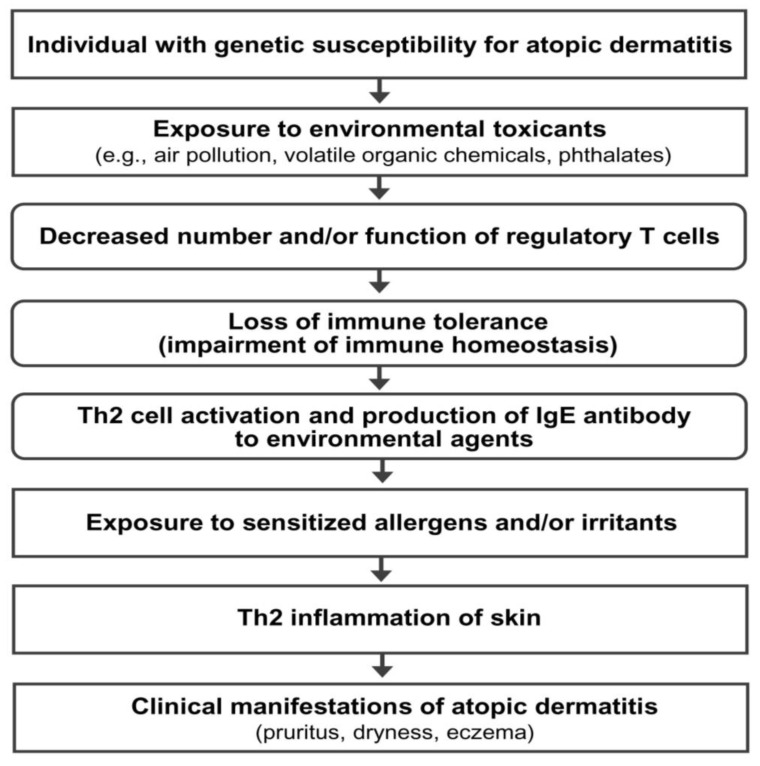
Hypothetical pathogenesis model for atopic dermatitis. Th2 cells, T-helper type 2 cells; IgE, immunoglobulin E.

AD is a multifactorial disorder caused by multiple pathogenic elements, including genetic predisposition, environmental triggers, immune dysfunction, hypersensitivity reaction, chronic skin inflammation, and skin barrier defect [2,3]. Unfortunately, the precise interactions among the multiple pathogenic elements involved in the development and maintenance of AD are not fully understood. Epidemiological and experimental evidence suggests that an impairment of immune tolerance state caused by decreased number and/or function of Treg cells resulting from the exposure to various environmental toxicants is responsible for a rapidly increased prevalence of allergic diseases in the industrialized world [3,11,12,13,14]. In this hypothetical pathogenesis model for AD, a human subject with a genetic predisposition for the development of AD is exposed to environmental toxicants (such as air pollution, volatile organic chemicals, phthalates, and bisphenol A) through the respiratory mucosa, gastrointestinal mucosa, or skin. The exposure to environmental toxicants decreases the number and/or function of Treg cells and impairs immune homeostasis (loss of immune tolerance state) in the subject. The toxicant-induced decreased number and/or function of Treg cells induces hypersensitivity in the human subject through the activation of Th2 cells and the development of an IgE response to common environmental agents. Exposure to sensitized allergens and/or chemical irritants induces a hypersensitivity reaction and chronic inflammation of the skin, which induces and maintains the clinical manifestations of AD (pruritus, dryness, and eczema) (Figure 1).

Based on the above hypothesis on AD pathogenesis, a decreased number and/or function of Treg cells suppressing IgE and Th2 cells plays a critical role in AD pathogenesis. Therefore, immunomodulatory strategies that activate Treg cells may be a useful therapeutic approach for inducing an LTCI or long-term treatment-free clinical remission of AD (disease-modifying therapy) by induction of immune tolerance (recovery of immune homeostasis).

## 4. Previous Reports on the Long-Term Clinical Improvement of AD

Previous reports have suggested that an LTCI of AD can be achieved through three methods. First, a marked change in the living environment, also known as climatotherapy (relocation of a patient to a different region with a beneficial climate such as a foreign country with favorable weather and clean air), has been reported to induce an LTCI of AD [15,16]. However, the LTCI induced by a marked change in the living environment usually disappears shortly after the patient returns to their previous environment, indicating that change in environmental factors alone cannot normalize the disordered immune system of patients with AD [15,16]. Second, allergen immunotherapy induces an LTCI in some patients with AD [17,18]. Multiple clinical studies have reported an LTCI of AD after allergen immunotherapy [17,18,19,20]. Third, up to 70% of children with AD experience a natural LTCI of AD before puberty [21]. Induction of immune tolerance (recovery of immune homeostasis) is the most probable mechanism of an LTCI observed in children with AD [3], although scientific studies could not directly demonstrate this hypothesis. Mimicking the immunological mechanism responsible for the natural LTCI of AD in children (induction of immune tolerance) may be an ideal therapeutic approach for achieving an LTCI of AD [3].

## 5. Five Questions and Three Hypotheses on the Regulatory T Cell-Targeted Immunomodulatory Strategies to Achieve a Long-Term Clinical Improvement of AD

The present author developed five key questions on the immune mechanisms that induce natural LTCI in children with AD and the LTCI observed after allergen immunotherapy in patients with AD (Table 1). Additionally, the present author proposes three working hypotheses and perspectives on immunomodulatory strategies to achieve an LTCI of AD (Table 2). These hypotheses and perspectives suggest that various immunomodulatory strategies activating Treg cells can induce an immune tolerance and achieve an LTCI in patients with AD.

## 6. Mechanism of Immune Tolerance and Rationale of Regulatory T Cell-Targeted Immunomodulatory Therapy for AD

Immune tolerance has been historically defined as a state of unresponsiveness of the immune system to self-antigens and foreign antigens (e.g., proteins and allergens) [22,23]. Prior exposure to a specific antigen has been suggested to induce immune tolerance to the antigen [22,23]. Immune tolerance is crucial for normal physiology, and defects in immune tolerance can lead to autoimmune and allergic diseases [24,25]. However, the present author prefers the term “immune homeostasis” because it is more scientifically appropriate than “immune tolerance” for several reasons. First, “immune tolerance” can be misinterpreted as having no immune response to self or foreign antigens. A well-controlled immune response (immune homeostasis) can maintain a harmoniously controlled immune response in contrast to an exaggerated immune response that can be harmful to the host. A low-grade, well-controlled autoimmune response to a self-antigen is physiological, and this response helps to remove denatured or altered self-antigens [26]. Well-controlled immune responses to microbial organisms also protect the body from severe infections (septicemia and viremia) or hyperactivation of the immune system. Hyperactivation of the immune system induced by viral infection causes uncontrolled systemic inflammation, cytokine release (cytokine storm), multi-organ failure, and death in the coronavirus disease 2019 [27]. Treg cells are a functionally defined subpopulation of T cells that modulate the immune system and play a critical role in immune homeostasis (a well-controlled immune response to self-antigens and foreign antigens), thereby preventing the development of autoimmune diseases, allergic diseases, and allograft rejection [28,29]. Animal experiments have suggested that decreased number and/or function (deficiency or dysfunction) of Treg cells serves as a key immune abnormality responsible for the development of autoimmune and allergic diseases [30].

Treg cells are classified as natural Treg cells (nTreg cells) and induced Treg cells (iTreg cells) [31]. nTreg cells express forkhead box P3 (Foxp3), CD4, and CD25 markers [32,33,34]. nTreg cells mediate “central immune tolerance” by deleting autoreactive lymphocyte clones before they develop into fully immunocompetent cells during lymphocyte development in the thymus [35]. Peripheral immune tolerance is mediated by iTreg cells and develops after T cells mature and enter the peripheral tissues and lymph nodes [36]. In peripheral immune tolerance, the immune response to a certain antigen can also be decreased by repeated antigenic exposure or by antigenic exposure in tolerogenic conditions [36]. iTreg cells arise extra-thymically from conventional (or naïve) CD4^+^ helper T cells in the presence of transforming growth factor-β (TGF-β), retinoic acid, and T cell receptor (TCR)-mediated antigen presentation by antigen-presenting cells (dendritic cells (DCs) or macrophages) in the peripheral tissue or nearby lymphoid tissue [31]. Among the iTreg cells, the IL-10-producing CD4^+^ Treg cells without Foxp3 expression (type 1 Treg cells: Tr1 cells) play a key role in allergen tolerance and can be induced by allergen immunotherapy in humans [37,38,39,40]. Previous studies have indicated that allergen-specific Tr1 cells are the predominant type of T cell response to allergens in healthy individuals and prevent unwanted hypersensitivity reactions to environmental antigens, such as house dust mites, pollen, and food [41,42]. There are significant differences in the proportions of three different allergen-specific T cell subtypes (T-helper type 1: Th1, Th2, and Tr1 cells) in peripheral blood between healthy non-allergic human subjects and allergic individuals [42]. The imbalance in the ratio of allergen-specific Th1, Th2, and Tr1 cells appears to be critical in the development of allergic diseases, and recovery of balance among allergen-specific Th1, Th2, and Tr1 cells may provide remission of allergic diseases, including AD [42]. Therefore, immunomodulatory approaches activating Tr1 cells with antigen-specific and/or antigen-nonspecific stimulations could induce an LTCI of AD by inducing immune homeostasis (immune tolerance state).

The presence of inborn errors of immunity (IEI) caused by genetic mutations that affect the immune system is important scientific evidence supporting the hypothesis that immune dysfunction resulting from a decreased number and/or function of Treg cells is critical in the pathogenesis of allergic diseases, including AD [30,43]. IEI are clinically expressed as increased susceptibility to infections, autoimmunity, allergy, bone marrow failure, and/or malignancy [44]. In human subjects, mutations of the Foxp3 gene (a master control gene of Treg cells) resulting in absent or dysfunctional Treg cells are responsible for immune dysregulation, polyendocrinopathy, enteropathy, X-linked (IPEX) syndrome, which is clinically characterized by neonatal autoimmune enteropathy, diabetes and thyroiditis, food allergies, and skin eczema [43,45]. AD is a frequent clinical manifestation observed in IPEX syndrome [43,45]. Another example is Wiskott–Aldrich syndrome (WAS) [46,47]. WAS is a rare X-linked recessive disease characterized by eczema, thrombocytopenia, immune deficiency, and bloody diarrhea [43]. WAS is caused by the genetic defects producing defective proteins (WAS proteins) that have a central role in actin polymerization and cytoskeletal rearrangement. Both nTreg cells and iTreg cells are defective in WAS [46]. In the WAS, the eczematous eruption is indistinguishable from AD when diagnostic criteria for AD are used and clears dramatically after a successful transplantation of bone marrow (hematopoietic stem cells) from a healthy donor [47]. This evidence from IEI demonstrates that Treg cell dysfunction is critically involved in the pathogenesis of the AD [30,43].

## 7. Immunomodulatory Strategies Activating Regulatory T Cells for AD: In Vivo Activation 

The Treg cell-targeted immunomodulatory therapies for AD include allergen immunotherapy, microbiota, vitamin D, polyvalent human immunoglobulin G (IgG), monoclonal antibodies to the surface antigens of T cell or antigen-presenting cell, and adoptive transfer of autologous Treg cells or genetically engineered Treg cells expanded in vitro (Table 3).

### 7.1. Allergen Immunotherapy (Activation of Allergen-Specific Tr1 Cells)

Allergen immunotherapy is a therapeutic approach that repeatedly administers a high dose of sensitized allergens to patients with allergic diseases either through subcutaneous injection or sublingual absorption to induce allergen-specific immune tolerance [48]. Based on a meta-analysis of multiple randomized clinical trials, allergen immunotherapy has been shown to be clinically beneficial in patients with AD sensitized to inhalant allergens such as house dust mites [49,50]. Allergen immunotherapy decreases allergic reactions (Th2 cell activation and IgE-mediated reaction) and induces clinical improvement in patients with allergic diseases through the activation of allergen-specific Treg cells [37,38,39,40]. Tr1 cells induced in the peripheral tissue and lymph nodes after repeated administration of a high dose of an allergen play a key role in the development of allergen-specific immune tolerance and clinical improvement of allergic diseases [37,38,39,40]. The allergen-specific Tr1 cells induced by allergen immunotherapy downregulate allergic inflammation by releasing IL-10 and TGF-β, which suppress the proliferation of allergen-specific Th2 cells and allergen-specific IgE production by B cell and promote the production of protective allergen-specific IgG antibodies [51]. Allergen immunotherapy increases the number of allergen-specific Tr1 cells [52]. However, it is unclear how Tr1 cells are induced during allergen immunotherapy [53]. A recent single-cell analysis study using allergen-specific T cell clones obtained from patients with allergic rhinitis suggested that allergen-specific Treg cells differentiate from allergen-specific Th2 cells during sublingual immunotherapy [54].

A recent systematic review and meta-analysis of 23 randomized, controlled trials, including 1957 patients, on allergen immunotherapy for AD showed that subcutaneous and sublingual immunotherapy to aeroallergens, particularly house dust mites, can significantly reduce AD severity and improve the quality of life [50]. However, this review also reported an increased risk of adverse events with subcutaneous immunotherapy compared to sublingual immunotherapy [50]. It took months (median five months) for allergen immunotherapy to provide clinical improvement in patients with AD sensitized to aeroallergens, and the probability of a marked reduction in the clinical severity of AD (50% or more from the baseline) was observed in 40% of the patients who received allergen immunotherapy and 26% of those who did not receive allergen immunotherapy [50]. In 1960, Tuft reported that subcutaneous immunotherapy using inhaled allergens in 101 patients with AD provided a favorable clinical response in 77.2% of the patients [20]. Here, clinical exacerbation of AD was observed in 5.0% (5/101 patients) of patients within 12 to 48 h after subcutaneous immunotherapy (“delayed adverse reaction”), and four of the five patients discontinued the allergen immunotherapy [20]. The risk of systemic adverse effects (anaphylaxis and delayed exacerbation of AD), relatively low success rate (<50%), and late-onset clinical efficacy (several months) are three major reasons that preclude the clinical application of allergen immunotherapy for the treatment of AD in real clinical practice.

### 7.2. Microbial Therapy

Probiotics, which are non-invasive, non-pathogenic bacteria with known health-promoting effects, are primarily found in fermented foods [55,56]. Intake of an adequate quantity of probiotics can be helpful for the homeostasis of the gut and immune system [57]. Probiotics can prevent AD in mice and humans [58,59,60]. *Lactobacillus* supplementation activates Treg cells in mouse models of allergy [61,62]. In recent systematic reviews and meta-analyses, probiotic strains composed of a mixture of multiple bacterial strains, including *Lactobacillus* and *Bifidobacterium*, were shown to provide significant clinical improvements in both pediatric and adult patients with AD [63,64] and reduce the risk of developing AD when administered to pregnant women, infants, or both [65]. However, there is controversy about the clinical efficacy of probiotics in the treatment of AD because there also have been systematic reviews and meta-analyses showing limited effectiveness of probiotics in the treatment of AD [66,67]. Further clinical trials are needed to evaluate the clinical usefulness of probiotics in the treatment of AD.

Prebiotics are non-digestible food ingredients (mainly carbohydrates and fibers) that promote the growth of healthy gut microflora by creating a nutrient-rich intestinal environment in which “good bacteria” may thrive. These beneficial bacteria in the large intestine, mainly *Lactobacillus* and *Bifidobacterium*, selectively ferment carbohydrates and fibers [68]. This process increases the number of commensal bacteria and the amount of acidic fermentation products, such as lactate and unbranched short-chain fatty acids (SCFAs, mainly acetate, butyrate, and propionate). Prebiotics can indirectly influence the immune system in patients with AD by supporting the growth of probiotics that produce SCFAs [69]. SCFAs exert anti-inflammatory effects in the intestine by acting on intestinal epithelial cells and facilitating the generation of iTreg cells [69]. A randomized, controlled trial which included 29 infants with AD reported significant clinical improvement after treatment with prebiotics (kestose) for 12 weeks [70]. Due to the lack of well-controlled, randomized clinical trials on prebiotics for AD, further investigations are needed [71]. Fecal microbiota transplantation (FMT) is the process of transferring fecal bacteria and other microbes from a healthy individual into another individual to restore the gut microbiota of a diseased individual. FMT increases the secretion of IL-10 from CD4^+^ T cells in mice [72]. FMT also reduced the clinical severity of AD and restored the Th1/Th2 ratio in an AD mouse model [73]. In an uncontrolled pilot clinical trial, FMT performed four times for six weeks (from week 4 to week 10) induced significant clinical improvement (decrease in clinical severity score of AD by more than 75% from the baseline) at week 18 in six (66.7%) out of nine adult patients with moderate-to-severe AD that had not been improved by prior topical and systemic therapies [74].

### 7.3. Vitamin D

Vitamin D increases the number and function of Treg cells [75,76,77]. DCs treated with active vitamin D (1,25-dihydroxycholecalciferol, also called calcitriol) become “tolerogenic” for the T cells, and calcitriol treatment induces the expression of the inhibitory cell-surface molecule programmed death ligand-1 on DCs and induces the differentiation of Treg cells from naïve T cells [78]. Calcitriol also acts directly on human CD4^+^ T cells, promoting the development of an IL-10-secreting CD4^+^ Treg cell population [79,80,81]. In BALB/c mice, dietary vitamin D3 supplementation significantly inhibited dinitrofluorobenzene-induced ear swelling and increased the number and suppressive activity of Treg cells in draining lymph nodes [82]. A recent systematic review of randomized, clinical trials on oral supplementation of vitamin D showed a significant clinical benefit in patients with AD [83].

### 7.4. Polyvalent Human IgG from Multiple Healthy Blood Donors

Intravenous administration of polyvalent human IgG, purified from the plasma pool of multiple healthy voluntary blood donors, has primarily been used to treat patients with primary immunodeficiency diseases associated with decreased immunoglobulin production [84]. Owing to its immunomodulatory effects, this therapy has also been used for the treatment of various autoimmune and allergic diseases [85,86]. In vitro incubation of polyvalent human IgG with purified CD4^+^CD25^high^ T cells increased the expression of intracellular IL-10, suggesting direct activation of Treg cells by polyvalent human IgG [87]. Intravenous administration of heterologous polyvalent human IgG induced a significant expansion of nTreg cells (CD4^+^CD25^+^Foxp3^+^) in patients with autoimmune diseases, including immune thrombocytopenia and eosinophilic granulomatosis with polyangiitis [88,89]. Therefore, Treg cell activation seems to be one of the major mechanisms responsible for the immunomodulatory and anti-inflammatory effects of polyvalent IgG [87,90,91]. However, clinical trials investigating the clinical efficacy of high-dose intravenous polyvalent IgG therapy in adult patients with severe AD did not show a significant clinical benefit [92]. The clinical efficacy and cost-effectiveness of high-dose intravenous polyvalent human IgG therapy for AD has not been completely evaluated yet. In contrast, subcutaneous administration of a mixture of histamine and polyvalent IgG (“Histaglobin”) significantly reduced the clinical severity of AD in a multicenter randomized, double-blind, controlled study [93]. Intramuscular administration of placenta-derived polyvalent IgG (“Allergobulin”) also significantly reduced the clinical severity of AD in a randomized, double-blind, controlled study [94]. The reason for the discrepancy in the clinical efficacy of polyvalent human IgG therapies for AD according to the routes of administrations (intravenous compared to subcutaneous or intramuscular) should be further evaluated in future studies.

### 7.5. Intramuscular Administration of Autologous Polyvalent IgG

“Jerne’s idiotypic network theory” (Jerne, 1974) proposes that antigen-binding sites (idiotypes) of autologous immunoglobulins are sufficiently immunogenic to induce antibody responses (anti-idiotype antibodies) in the same host [95,96]. Induction of an anti-idiotypic immune response has been suggested as one of the major mechanisms responsible for the development of immune tolerance (immune homeostasis) [97]. Physiological idiotype-anti-idiotype antibody responses maintain immune homeostasis by controlling excessive immune responses to self or foreign antigens [96,97,98]. Stimulation of anti-idiotypic immunomodulation has been suggested as a promising therapeutic approach for allergic diseases [99]. However, there is scarce evidence supporting the clinical efficacy of anti-idiotypic immunomodulatory therapy in human subjects with allergic diseases. The present author hypothesized that intramuscular administration of autologous total IgG could induce an anti-idiotypic immunomodulatory effect and clinical improvement in patients with allergic diseases [100,101,102,103,104,105]. To prove this concept, the clinical efficacy, safety, and immunomodulatory effects of intramuscular administration of autologous total IgG were evaluated in a randomized, double-blind, placebo-controlled study involving 51 adolescent and adult patients with moderate-to-severe AD [104]. In this study, eight weekly intramuscular administrations of 50 mg autologous total IgG for seven weeks significantly decreased the clinical severity of AD and increased serum levels of IL-10 and interferon (IFN)-γ at weeks 4, 8, 12, and 16 compared to the baseline without serious adverse events [104]. These results showed that intramuscular administration of autologous total IgG could provide clinical improvement and systemic immunomodulatory effects in patients with AD. To further evaluate the mechanism of immunomodulation induced by intramuscular administration of autologous total IgG, changes in peripheral blood T cells were analyzed before and after administration in 13 healthy human subjects [105]. Intramuscular administration of autologous total IgG significantly increased the percentage of IL-10-producing CD4^+^ T cells and the percentage of IFN-γ-producing CD3^+^ T cells in healthy human subjects. These results suggest that intramuscular administration of autologous total IgG activates Tr1 cells in healthy human subjects [105]. Interestingly, long-term clinical improvements and decreases in serum total IgE concentrations lasting for more than 36 weeks were observed in two of three patients with severe AD who were followed up for more than two years after eight intramuscular administrations of 50 mg autologous total IgG for four weeks [102].

Major limitations of previous studies on the clinical efficacy and immunomodulatory efficacy of intramuscular administration of autologous total IgG in patients with AD and healthy human subjects include the lack of knowledge on the detailed molecular mechanism of Treg cell activation induced by intramuscular administration of autologous total IgG. An in vitro study showed that pooled IgG purified from AD patients induced significantly higher productions of IL-10 and IL-17 from cultured thymic CD4^+^ T cells than pooled purified IgG from healthy blood donors (polyvalent IgG for intravenous administration) [106]. Other in vitro experiments showed that pooled IgG purified from AD patients induced significantly higher productions of IFN-γ and IL-22 from cultured thymic gamma-delta T cells compared to pooled purified IgG from healthy blood donors [107]. These experimental results suggest that polyvalent IgG from AD patients might more efficiently activate Treg cells than polyvalent IgG from healthy blood donors by direct interactions between idiotypes of IgG and T cells without the help of antigen-presenting cells (“hook without bait theory”) as previously proposed [108]. However, further studies are necessary to evaluate detailed immunological mechanisms producing a therapeutic efficacy of intramuscular administration of autologous total IgG in patients with AD.

### 7.6. Monoclonal Antibodies to Antigens on the Surface of T Cells or Antigen-Presenting Cells

OX40 (CD 134) is expressed only in activated T cells, and its ligand OX40 ligand (OX40L, CD252) is expressed only on activated antigen-presenting cells [109]. OX40L binds to OX40 on T cells and prevents activated T cells from dying, subsequently increasing cytokine production. OX40 plays a critical role in the maintenance of an immune response beyond the first few days, enhances the survival of activated T cells, and plays a crucial role in both Th1- and Th2-mediated reactions in vivo [110]. OX40 and OX40L are only induced after antigen or TCR stimulation, and the OX40–OX40L interaction is a key regulator of T cell responses [111]. OX40 signaling can promote effector T cell proliferation and inhibit Treg cell function [109]. OX40 has been suggested as a potential immunotherapeutic target for cancer, inflammatory, and autoimmune diseases [109]. Blocking the OX40–OX40L interaction and OX40 signaling enhanced Treg cell proliferation under in vitro conditions; this approach has been suggested as a novel strategy to increase Treg cells and suppress autoimmunity [112]. An agonist anti-OX40 monoclonal antibody induced Treg cell expansion in a mouse experimental model [113]. In a non-human primate graft versus host disease model using the transplantation of allogeneic hematopoietic stem cells, concurrent blockade of OX40L with monoclonal antibody (KY1005) and mechanistic target of rapamycin (mTOR) with sirolimus increased Treg cells in the blood and prevented graft rejection [114]. In a multicenter, double-blind, placebo-controlled phase 2b clinical study, an anti-OX40 antibody (rocatinlimab) provided significant clinical improvement in patients with moderate-to-severe AD [115]. Interestingly, a significant proportion of patients who achieved marked clinical improvement (achieving at least a 75% reduction in the clinical severity score of AD from the baseline) after anti-OX40 antibody therapy for 36 weeks experienced prolonged clinical improvement for more than 20 weeks after discontinuation of the therapy [115]. Further clinical studies on the possibility of inducing an LTCI of AD by anti-OX40 antibody therapy are needed.

### 7.7. Other Chemical Candidates for Regulatory T Cell-Targeted Therapy of AD

There are several other chemicals (small molecules) that have been shown to activate Treg cells, but their immunomodulatory effects and clinical efficacy in patients with AD have not been evaluated yet.

#### 7.7.1. Sirolimus

Sirolimus (also known as rapamycin) is a macrolide compound with immunosuppressant activity that has been used to prevent organ transplant rejection [116]. It is an inhibitor of the mTOR and inhibits the activation of T and B cells by reducing their sensitivity to IL-2 [117]. Human Treg cells expand efficiently in the presence of sirolimus under experimental cell culture conditions [118]. Sirolimus also provided major clinical improvements and expansion of peripheral blood Treg cells in patients with systemic lupus erythematosus [119]. Topical application of sirolimus ointment reduced the clinical severity of eczema in an AD mouse model [120].

#### 7.7.2. Metformin

Metformin is a first-line therapeutic agent for type 2 diabetes [121]. It activates adenosine monophosphate-activated protein kinase, which inhibits mTOR complex1, controlling the activation and differentiation of T and B cells, thereby producing immunomodulatory and anti-inflammatory effects [121]. Oral administration of metformin improved autoimmune arthritis in a mouse model of collagen-induced arthritis [122]. In the collagen-induced arthritis mouse model and in vitro experiments, metformin treatment decreased Th17 cell differentiation and enhanced Treg cell differentiation [122,123]. In a randomized, double-blind, placebo-controlled clinical trial, patients with active rheumatoid arthritis receiving continuous methotrexate therapy were further treated with additional metformin (1000 mg/day) or placebo [124]. After 12 weeks of treatment, metformin-treated patients showed better clinical outcomes and higher remission rates than placebo-treated patients [124]. Oral administration of metformin provided a significant attenuation of skin inflammation in the AD mouse model [125].

#### 7.7.3. Butyrate

Butyrate is one of the SCFAs found in animal fat and plant oils, bovine milk, breast milk, butter, parmesan cheese, body odor, vomit, and as a product of anaerobic fermentation in the colon [126,127,128]. Highly fermentable fiber residues (e.g., resistant starch, oat bran, pectin, and guar) are transformed by colonic bacteria into SCFAs including butyrate [128,129]. Butyrate produced in the colon plays a key role in maintaining immune homeostasis both locally (in the gut) and systemically (via circulating butyrate) [130]. Butyrate promotes the differentiation of Treg cells [130,131] and is the most potent promoter of intestinal Treg cells among the various SCFAs [131]. In patients with ulcerative colitis, an enema with butyrate decreased inflammation and bleeding [132]. In an AD mouse model, oral intake of sodium butyrate alone or in combination with probiotics increased serum levels of IL-10 and mitigated AD symptoms [133].

### 7.8. Adoptive Cell Therapy with Ex Vivo Expanded Regulatory T Cells

Recent technical developments have enabled adoptive cell therapy (ACT) with ex vivo expanded Treg cells. ACT with Treg cells can be achieved using three methods: polyclonal Treg cells without antigen stimulation, antigen-stimulated and expanded Treg cells, and Treg cells with a genetically engineered chimeric antigen receptor. Adoptive transfer of Treg cells has been proposed to treat T cell-mediated immune diseases including organ transplant rejection, autoimmune diseases, and allergic diseases [134,135]. However, human clinical studies using the adoptive transfer of ex vivo expanded Treg cells for AD or other allergic diseases have not been conducted. Future studies on the clinical efficacy and immunomodulatory effects of the adoptive transfer of ex vivo expanded Treg cells in patients with AD or animal models of AD are needed.

#### 7.8.1. Autologous Polyclonal Regulatory T Cells

Treg cells can be isolated from the peripheral blood of human subjects and cultured in vitro to expand [136]. In a human clinical trial involving 12 patients with type 1 diabetes (autoimmune diabetes), intravenous infusion of autologous nTreg cells (CD4^+^CD25^+^ Foxp3^+^ T cells purified from patients’ peripheral blood and expanded in vitro using beads coated with anti-CD3 and anti-CD28 antibodies, IL-2, and autologous serum) resulted in a significant decrease in the requirement of exogenous insulin (in 8 of 12 patients) compared to the untreated control group (in 2 of 10 patients) without significant adverse events [137]. In a human clinical trial for kidney transplantation, adoptive cell transfer of autologous Treg cells by intravenous infusion was effective and well-tolerated [138]. In a phase I/IIa clinical trial investigating Treg cell therapy in kidney transplantation, patients engrafted with autologous Treg cells had similar rejection rates compared to the control group receiving the standard immunosuppression with basiliximab (anti-CD25) but displayed reduced infection rates [138]. In another human trial for patients with kidney transplantation, a single-dose intravenous infusion of autologous nTreg cells was performed seven days after kidney transplantation in 11 patients undergoing living-donor kidney transplantation [139]. Stepwise tapering of triple immunosuppression (prednisolone, mycophenolate, and tacrolimus) to low dose tacrolimus monotherapy was attempted until week 48. Stable monotherapy immunosuppression (without prednisolone) was achieved in eight of 11 (73%) patients that received nTreg cells, while the control group remained on standard dual or triple drug immunosuppression (with continuous prednisolone) (*p* = 0.002). Both the nTreg cell treatment and control groups had 100% three-year allograft survival and similar clinical and safety profiles [139]. In future clinical or preclinical studies on AD, it will be possible to conduct a study in which autologous nTreg cells derived from the peripheral blood of patients with AD are expanded in vitro and subsequently re-injected into the same patients.

#### 7.8.2. Autologous Antigen-Stimulated Regulatory T Cells

Theoretically, an ACT with an antigen-specific approach might be more efficient with higher specificity than polyclonal Treg cells without antigen stimulation [140]. Treg cells obtained from kidney allograft recipients can be stimulated by antigen-presenting cells from the kidney graft donor to generate autologous Treg cells reactive to donor alloantigens [141]. Antigen-specific Tr1 cells can be enriched in cultured CD4^+^ T cells that were stimulated with allogeneic IL-10-producing DCs generated from CD14^+^ monocytes (from the kidney donor) in the presence of IL-10 [142]. These allospecific Tr1 cells showed specific immune suppression to donor antigens (alloantigens) in vitro and maintained a tolerogenic gene expression profile in vivo [142]. However, human clinical studies in kidney transplantation with intravenous administration of donor alloantigen-stimulated autologous Treg cells resulted in significant rejection risk following immunosuppressive drug weaning [143]. Further developments are needed before antigen-specific Treg cell therapy can be applied clinically. In future clinical or preclinical studies of AD, autologous Treg cells derived from patients with AD sensitized to allergens can be cultured and expanded with allergens (e.g., house dust mite allergens or cat allergens) in vitro and re-infused into patients with AD.

#### 7.8.3. Genetically Engineered Regulatory T Cells

Genetic modification of polyclonal Treg cells to express chimeric antigen receptor (CAR) can be used to recognize a specific antigen, thereby providing antigen-specific Treg cells [144]. The advantage of genetically engineered Treg cell therapy is the reduction in side effects due to non-specific immune suppression by Treg cells [145]. Treg cells can be isolated from the peripheral blood of healthy and allergic donors, cultured with IL-2 for polyclonal Treg expansion, and then transformed into genetically engineered Treg cells by transduction with retroviruses, lentiviruses, or adenovirus to express CAR. Following Good Manufacturing Practice regulations, expanded cells can be infused into patients [146]. CAR is a single protein complex consisting of a single chain antibody fragment that recognizes the antigen in the outer part, a hinge, and an inner part with CD3ζ and CD28/4-1BB domains.

The use of CD8^+^ cytotoxic T cells bearing antigen-specific CAR, designed to redirect T cells from cancer patients to antigen-expressing tumor cells, is the main ACT strategy for cancer therapy [146,147]. The first CAR-T cell therapy approved by the Food and Drug Administration is tisagenlecleucel (CTL019; trade name Kymriah). Tisagenlecleucel is a CD19-directed genetically modified autologous T cell immunotherapeutic agent for the treatment of adult patients with relapsed or refractory follicular lymphoma and B cell precursor acute lymphoblastic leukemia [148]. CAR-T cell therapy for hematological malignancies has been primarily designed to recognize the CD19 antigen on the surface of B cells, including normal lymphocytes and leukemic cells. CD19 was chosen as a target for immunotherapy because it is uniformly expressed in B cell leukemia/lymphomas and healthy B cells but not in other normal tissues [149]. The production of tisagenlecleucel involves reprogramming the patient’s own T cells with a transgene encoding CAR to identify and eliminate CD19-expressing cells. Upon binding to CD19-expressing cells, the CAR transmits a signal that promotes T cell expansion [150]. The major advantage of CAR-T cells is their ability to be human leukocyte antigen-independent. Obviating the need for antigen presentation in human leukocyte antigen makes this technology more accessible and universal [151]. CAR-engineered T cell therapy is becoming the most promising approach in cancer treatment, involving reprogramming of the patient’s own T cells with a CAR-encoding transgene to identify and eliminate cancer-specific surface antigen-expressing cells. However, the adverse effects of CD8^+^ cytotoxic CAR-T cells are severe and are characterized by over-activation of the immune system, including cytokine release syndrome, encephalopathy syndrome, hemophagocytic lymphohistiocytosis, tumor lysis syndrome, B cell aplasia, and acute respiratory distress syndrome [152,153,154]. Major trials have indicated that the incidence rate of cytokine release syndrome was 77% in patients treated for acute lymphoblastic leukemia [155] and 57–93% in non-Hodgkin lymphoma patients [156,157].

Recently, the CAR approach was evaluated in an ovalbumin (OVA) allergy mouse model. In this study, the CAR Treg cell was composed of OVA linked with the transmembrane and signal transduction domains, CD28-CD3ζ [158]. This CAR Treg cell therapy decreased the anaphylactic reaction induced by intraperitoneal OVA injection in the mouse model [158]. Under cell culture conditions, Treg cells transduced with Bet v 1-specific TCR suppressed allergen-specific effector T cell proliferation and cytokine production [159].

#### 7.8.4. Limitations of Adoptive Cell Therapy with Regulatory T Cells

Human Treg cells display phenotypic plasticity and can be changed into effector T cells (Th1, Th2, or Th17 cells) in an inflammatory environment, and the effector cells produce pathogenic cytokines, such as IFN-γ, IL-4, IL-13, and IL-17 [160,161]. The instability of Treg cells is a crucial limiting factor for the successful development of ACT with Treg cells in immune diseases [162,163,164,165]. The isolation of low-frequency Treg cells from autologous peripheral blood cells is complex and expensive [166]. Currently, ACT with Treg cells has important limitations in its cost, clinical efficacy, and safety. Further technical improvements are needed to achieve a successful clinical application of ACT with Treg cells in AD.

## 8. Combinations of Different Modalities Activating Regulatory T Cells

**Figure 2:**
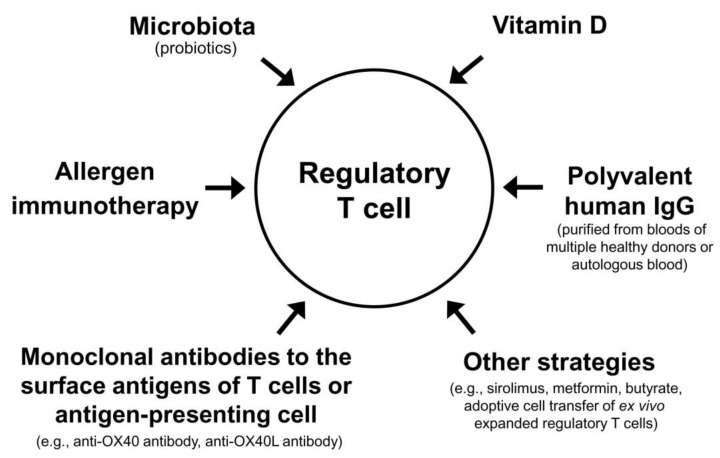
Regulatory T cell-targeted immunomodulatory strategies to achieve a long-term clinical improvement of atopic dermatitis by monotherapy or combination therapy.

Theoretically, combinations of currently available methods that can activate Treg cells (allergen immunotherapy, microbiota, vitamin D, polyvalent human IgG, and small molecular chemicals) may maximize the clinical improvement of patients with AD. High dose vitamin D3 enhanced the clinical efficacy and immunomodulatory effects of subcutaneous allergen immunotherapy in a grass pollen-driven mouse model of asthma [167]. In a randomized, controlled clinical trial including children with allergic rhinitis, vitamin D supplementation combined with grass pollen sublingual immunotherapy was more effective in reducing nasal and asthma symptoms than grass pollen sublingual immunotherapy alone [168]. In patients with allergic rhinitis and vitamin D deficiency, vitamin D supplementation in the build-up phase of subcutaneous allergen immunotherapy with house dust mite extract significantly decreased the symptom–medication score of allergic rhinitis compared to subcutaneous allergen immunotherapy alone [169]. In a previous double-blind, placebo-controlled, randomized clinical trial, repeated intradermal injections of immune complexes made of house dust mite antigens and autologous antibodies to house dust mite antigens produced significant clinical improvement in patients with AD [170]. These examples suggest that various combinations of different modalities activating Treg cells can improve a long-term clinical outcome of AD (Figure 2).

## 9. Conclusions

**Figure 3:**
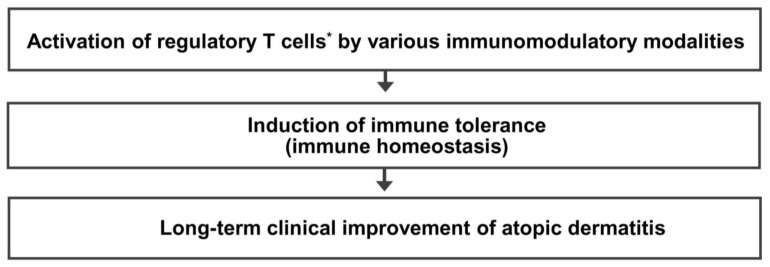
Regulatory T cell-targeted immunomodulatory therapies to achieve long-term clinical improvement of atopic dermatitis by induction of immune tolerance. The present author proposes a hypothesis that many different immunomodulatory strategies inducing a sufficient long-term activation of regulatory T cells can improve long-term clinical outcomes and provide a long-term treatment-free clinical remission of atopic dermatitis by induction of immune tolerance. * Activation of regulatory T cells means an increase in number and/or function of regulatory T cells.

Immune dysfunction resulting from a decreased number and/or function of Treg cells is critical in the pathogenesis of allergic diseases, including AD. Treg cell activation could be a common immune mechanism responsible for the LTCI observed in patients with AD after allergen immunotherapy and the natural clinical remission observed in children with AD. Therefore, an immunomodulatory strategy that activates Treg cells could be an ideal therapeutic approach to achieve an LTCI of AD. The present author proposes a hypothesis that many different immunomodulatory strategies inducing a sufficient long-term activation of Treg cells can improve the long-term clinical outcome and provide a long-term treatment-free clinical remission of AD by induction of immune tolerance (Figure 3). Further studies on the clinical efficacy of various Treg cell-targeted immunomodulatory therapies should be conducted to improve the long-term clinical outcome in patients with AD.

## Figures and Tables

**Table 1:** Five questions on the immune mechanism responsible for a long-term clinical improvement observed in patients with atopic dermatitis.

What is the mechanism of natural induction of a long-term clinical improvement of atopic dermatitis in children? Is the induction of immune tolerance responsible for a natural long-term clinical improvement of atopic dermatitis in children? Is the activation of type 1 regulatory T cells a key mechanism inducing a natural long-term clinical improvement of atopic dermatitis? Is the activation of type 1 regulatory T cells a common mechanism inducing a long-term clinical improvement of atopic dermatitis by allergen immunotherapy and a natural long-term clinical improvement in children with atopic dermatitis? Can various kinds of immunomodulatory strategies activating regulatory T cells provide a long-term clinical improvement in patients with atopic dermatitis?

**Table 2:** Three hypotheses and perspectives to achieve a long-term clinical improvement of atopic dermatitis by regulatory T cell activations.

A decreased number or function of regulatory T cells is a critical event that causes the activation of Th2 cells, leading to the development and maintenance of atopic dermatitis. Activation of regulatory T cells * is an effective therapeutic approach to achieve a long-term clinical improvement of atopic dermatitis. Many different immunomodulatory strategies activating regulatory T cells can provide a long-term clinical improvement of atopic dermatitis by induction of immune tolerance.

* Activation of regulatory T cells means an increase in the number and/or function of regulatory T cells. Th2 cells, T-helper type 2 cells.

**Table 3:** Immunomodulatory strategies activating regulatory T cells for the treatment of atopic dermatitis.

Strategies with proven clinical efficacy by at least one randomized clinical trial
(1) Allergen immunotherapy (2) Microbial therapy (probiotics) (3) Vitamin D (4) Subcutaneous or intramuscular injection of polyvalent human IgG from multiple healthy blood donors (5) Intramuscular injection of autologous total IgG (6) Monoclonal antibody to antigen on the surface of T cells (anti-OX40 antibody)
Strategies without proven clinical efficacy in patients with atopic dermatitis by a clinical trial
(1) Sirolimus (also called rapamycin) (2) Metformin (3) Butyrate (4) Adoptive cell therapy with ex vivo expanded regulatory T cells

IgG, immunoglobulin G.

## Data Availability

Not applicable.
